# Implementing a Hybrid Digital-Physical Health Dialogue Model in a Rural Japanese Community: A Technical Report

**DOI:** 10.7759/cureus.100393

**Published:** 2025-12-30

**Authors:** Ryuichi Ohta, Toshihiro Yakabe

**Affiliations:** 1 Community Care, Unnan City Hospital, Unnan, JPN; 2 Family Medicine, Unnan City Hospital, Unnan, JPN

**Keywords:** community health services, health communication, patient participation, program evaluation, rural health, telemedicine

## Abstract

Rural communities often face structural and psychological barriers to healthcare access, particularly among older and socially isolated populations. Conventional clinic-based services may not adequately address early, ambiguous health concerns or facilitate low-threshold help-seeking. This technical report describes the implementation of a hybrid digital-physical health dialogue model designed to enhance accessibility, trust, and sustained engagement in a rural Japanese community. The intervention integrated an anonymous, free health consultation service delivered via the LINE messaging platform (LINE Corporation, Tokyo, Japan) with face-to-face dialogue sessions conducted at local community centers. Healthcare professionals and a community-based organization collaboratively facilitated both components. Rather than evaluating effectiveness, this report focuses on the implementation process and on observational insights from routine operations, including staff field notes, informal debriefings, and participant feedback. The digital consultation service served as a low-threshold entry point, enabling residents to raise health-related concerns early. Over time, some participants transitioned from online consultations to in-person dialogues, which may indicate gradual trust-building and increased confidence in engagement. Face-to-face sessions appeared to foster mutual recognition, peer support, and a shared understanding of community health concerns. Healthcare professionals reported enhanced contextual awareness of residents’ lived experiences. Key challenges included digital exclusion among some older adults, reliance on voluntary human resources, and the absence of formal outcome evaluation. The hybrid digital-physical health dialogue model demonstrates practical feasibility as an implementation approach to support accessible and relational health dialogue in a rural setting. While not an effectiveness study, the findings provide transferable insights for designing community-based, low-threshold health dialogue initiatives that balance accessibility, trust, and sustainability in resource-limited contexts.

## Introduction

In rural Japan, disparities in access to healthcare and health-related information have become increasingly evident, particularly among older adults and socially isolated populations [1,2]. Geographic barriers, shortages of healthcare professionals, and limited public transportation restrict access to timely medical care, while social norms and stigma further discourage help-seeking behaviors. As a result, many residents experience prolonged uncertainty regarding their health concerns, potentially associated with preventable deterioration in physical and psychological well-being [1,2].

Recent studies have highlighted that older adults in rural settings often hesitate to seek formal medical care, relying instead on informal coping strategies or delaying consultation until symptoms worsen [1,2]. These patterns are not solely attributable to individual preferences but are shaped by structural factors, including limited access points to care, inadequate health information, and a lack of spaces where residents feel safe to voice concerns. Addressing these barriers requires approaches that extend beyond conventional clinic-based services and engage with the everyday contexts of rural life.

Digital health interventions have emerged as a potential means of mitigating access gaps by reducing geographical and psychological barriers to consultation. In rural Japan, online platforms have been shown to facilitate health-related dialogue, promote help-seeking behaviors, and create new communities of practice that connect residents with healthcare professionals [3,4]. However, digital approaches alone may be insufficient. Older adults may face challenges related to digital literacy, device access, and trust, underscoring the importance of complementary face-to-face interactions that foster embodied communication and long-term relationships [3,4].

To address these challenges, the Unnan Community Health and Development Lab (Machizukuri-koubo Unnan) implemented a hybrid model that integrates anonymous LINE (LINE Corporation, Tokyo, Japan)-based health consultations with in-person dialogues conducted at local community centers [3,4]. This model was designed to provide accessible entry points for health dialogue while preserving the relational and contextual strengths of face-to-face communication. By combining digital and physical spaces, the initiative sought to reduce informational inequities, support help-seeking behaviors, and strengthen trust between residents and healthcare professionals. Unnan City was selected as the implementation site because it represents a typical rural Japanese municipality characterized by rapid population aging, geographic dispersion, and long-standing collaboration between healthcare professionals and community organizations, providing a suitable context for piloting a practice-based, dialogue-oriented model.

Multidimensional perspectives were used to contextualize and interpret the conceptual design of this hybrid approach to social justice and human agency. Fraser’s framework provides a lens to understand issues of equitable participation through redistribution, recognition, and representation. At the same time, Sen's capability approach offers a way to interpret how the intervention may expand individuals' opportunities to engage in health-related dialogue and decision-making [5,6]. These frameworks were applied retrospectively to situate the intervention within broader theoretical discussions, rather than serving as prescriptive models guiding its initial design [5,6]. In the context of rural healthcare, these perspectives underscore the need to create systems that are both structurally fair and responsive to individuals’ lived experiences [7]. These frameworks informed the practical design of the intervention by emphasizing low-threshold participation (redistribution of access), respect for residents’ lived experiences (recognition), and the creation of multiple, flexible entry points for health dialogue (representation). The hybrid digital-physical model was therefore conceptualized not as a technological solution alone, but as a participatory structure aimed at expanding residents’ capabilities to engage in health-related dialogue according to their individual contexts.

The purpose of this technical report is to describe the implementation of a hybrid digital-physical health dialogue model in a rural Japanese community and to share practical insights gained through its operation. Rather than evaluating effectiveness through quantitative outcomes, this report focuses on the implementation process, observed impacts, and challenges encountered, to inform similar practice-based initiatives in other rural and resource-limited settings.

## Technical report

Context and problem statement

Unnan City is a rural municipality in Japan characterized by geographic dispersion, a rapidly aging population, and limited healthcare resources. The city was selected as the implementation site due to long-standing partnerships between healthcare professionals and community organizations, as well as its demographic characteristics that are representative of many rural Japanese communities [8]. As in many rural regions, residents face long travel distances to medical facilities, reduced public transportation options, and shortages of healthcare professionals, all of which constrain timely access to care [2]. These structural conditions disproportionately affect older adults, caregivers, and socially isolated individuals, who may already experience physical limitations or diminished social networks.

Within this context, help-seeking behaviors are often shaped by both practical and cultural barriers. Previous research has shown that older adults in rural settings tend to delay or avoid seeking medical consultation due to concerns about burdening others, fear of being perceived as complainers, and uncertainty about the legitimacy of their symptoms [1,2]. Such hesitancy is reinforced by limited opportunities for informal health dialogue, which may be associated with unmet health needs that remain invisible to formal healthcare systems.

Traditional clinic-based services alone are insufficient to address these challenges. While outpatient care remains central to rural healthcare delivery, it often fails to accommodate early, low-threshold consultations for ambiguous or non-acute concerns [1,2]. Residents with mild symptoms, psychological distress, or caregiving-related worries may lack appropriate entry points into the healthcare system, and such gaps in access have been associated with delayed diagnosis, potentially preventable disease progression, and increased anxiety [1,2]. These gaps led us to hypothesize that alternative platforms might enable residents to seek advice before their conditions escalate.

Digital health initiatives have shown promise in expanding access to health information and consultation in rural Japan. Online-based community dialogues and digital consultation platforms have been reported to support help-seeking behaviors, facilitate knowledge sharing, and reduce social isolation by connecting residents with healthcare professionals and peers [3]. However, reliance on digital solutions alone presents limitations. Older adults may encounter barriers related to digital literacy, device availability, and trust in online communication, which can reproduce existing inequities rather than resolve them [4].

Conversely, face-to-face community-based dialogues have been demonstrated to foster trust, mutual recognition, and sustained engagement in rural healthcare settings [4]. Such interactions allow residents to share concerns within familiar social spaces, reinforcing relational continuity and collective understanding [9]. Nevertheless, in-person initiatives are constrained by mobility, time, and staffing resources, limiting their reach to those who are already socially connected or physically able to attend.

Against this backdrop, a critical gap exists between accessibility and relational depth in rural health dialogue. Digital platforms can lower entry barriers but may lack the embodied trust necessary for sustained engagement, while face-to-face interactions provide rich relational contexts but are not universally accessible [10]. Addressing this tension was conceptualized by the authors as potentially benefiting from an integrated approach that bridges digital and physical spaces, creating flexible pathways for participation that accommodate diverse needs and capabilities.

The Unnan Community Health and Development Lab sought to respond to this challenge by developing a hybrid digital-physical health dialogue model that combines anonymous online consultations with community-based face-to-face interactions [3,4]. This approach was designed to provide multiple entry points for health dialogue, reduce psychological and geographic barriers, and support gradual engagement with healthcare professionals. The present technical report situates this implementation within the broader challenges of rural healthcare access and aims to clarify the contextual problems that motivated the development of the hybrid model.

Description of the intervention

**Figure 1:**
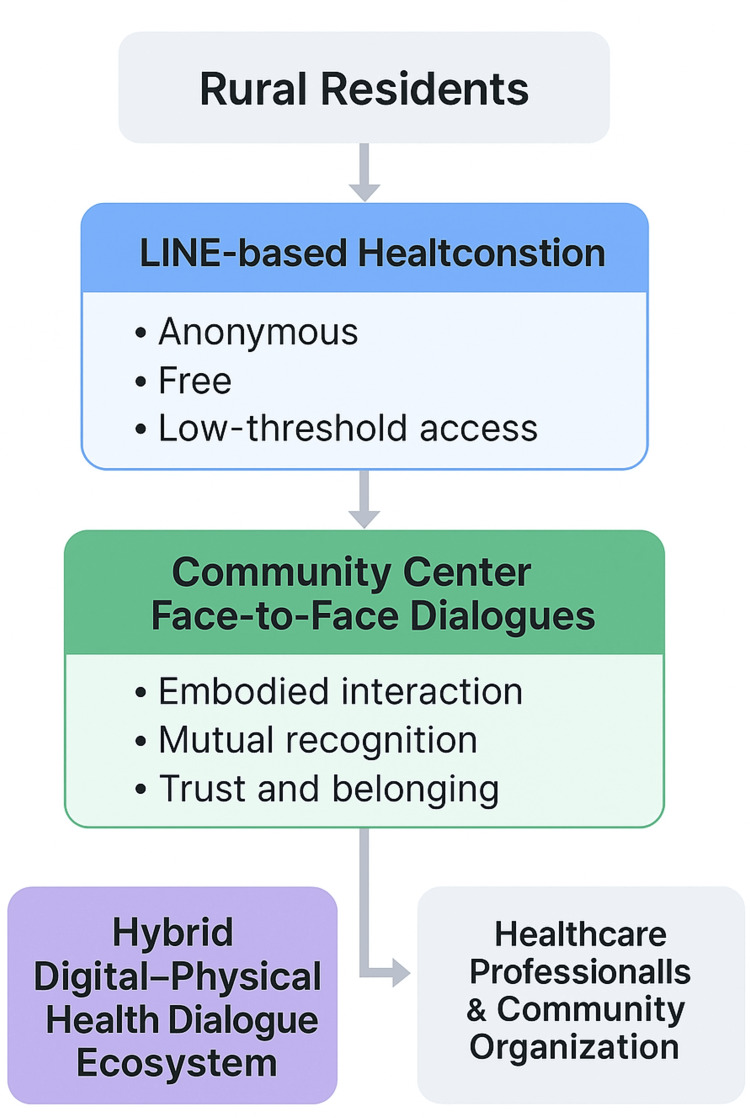
Conceptual framework of the hybrid digital-physical health dialogue model implemented in a rural Japanese community. This figure depicts the overall structure of a hybrid health dialogue ecosystem that integrates anonymous digital consultations delivered via the LINE messaging platform with face-to-face dialogue sessions conducted at local community centers. Rural residents may enter the system through either digital or physical pathways depending on their accessibility, preferences, and psychological readiness. Healthcare professionals and a community-based organization collaboratively support both components by facilitating dialogue, providing guidance, and maintaining continuity of engagement. The model emphasizes accessibility, trust-building, and relational continuity rather than clinical decision-making, illustrating how digital and physical spaces function as complementary, interconnected platforms within a single community-embedded system. Image credit: Ryuichi Ohta.

To address barriers to health access and dialogue in a rural context, the Unnan Community Health and Development Lab implemented a hybrid intervention integrating digital and face-to-face communication platforms [3,4]. The intervention was designed to provide multiple, flexible entry points for health-related dialogue, enabling residents to engage according to their individual circumstances, preferences, and capabilities. The core components of the intervention consisted of (1) an anonymous LINE-based health consultation service and (2) in-person community dialogue sessions held at local community centers (Figure 1).

LINE-Based Health Consultation

The digital component of the intervention was an anonymous health consultation service delivered through LINE, a widely used messaging application in Japan [3]. This service was offered free of charge and allowed residents to submit health-related questions without disclosing personal identifiers. Consultations addressed a broad range of concerns, including chronic symptoms, mental health issues, caregiving burdens, medication-related questions, and uncertainty about when to seek formal medical care.

Healthcare professionals affiliated with the Unnan City Hospital and the associated non-profit organization (NPO) responded to consultations, providing informational guidance, emotional support, and recommendations regarding appropriate help-seeking pathways when necessary [11]. The use of anonymity was a deliberate design choice, intended to help reduce psychological barriers associated with stigma, fear of burdening healthcare providers, or uncertainty about symptom legitimacy. By lowering these thresholds, the LINE platform was designed to serve as an accessible initial point of contact for individuals who might otherwise refrain from seeking assistance [3].

Community-Based Face-to-Face Dialogues

To complement digital consultations, the intervention incorporated regular face-to-face dialogue sessions conducted at community centers across Unnan City [4]. These sessions were organized in collaboration with local stakeholders and healthcare professionals and were open to residents of all ages and health statuses. The dialogues emphasized horizontal communication, encouraging participants to share experiences, concerns, and perspectives within a non-hierarchical setting, rather than through conventional clinician-patient interactions.

Face-to-face dialogues were designed to provide opportunities for embodied communication, mutual recognition, and trust-building, which we hypothesized might complement digital interactions rather than replace them [4]. Healthcare professionals participated as facilitators rather than authority figures, fostering an environment in which residents felt respected and validated. These sessions were also designed to serve as venues for collective reflection on community health challenges and to support the exploration of locally relevant solutions.

Integration of Digital and Physical Components

A defining feature of the intervention was the intentional integration of digital and physical dialogue spaces [3,4]. Rather than operating independently, the LINE consultation service and community-based dialogues were designed to function as interconnected components within a single participatory system. Some residents initiated engagement through anonymous digital consultations and later attended face-to-face dialogues, which may have reflected developing familiarity with the program rather than a formally measured change in trust or confidence. Conversely, participants who first encountered the program through community events frequently continued discussions via LINE, enabling ongoing access to support beyond physical meetings.

This bidirectional circulation between digital and physical spaces allowed the intervention to accommodate diverse participation patterns, including those constrained by mobility limitations, time availability, or social isolation. By maintaining continuity across platforms, the hybrid model supported sustained engagement while preserving accessibility and relational depth.

Design Principles

The intervention was guided by several key design principles: low-threshold access, anonymity and confidentiality, relational continuity, and community embeddedness. Digital tools were employed not as substitutes for in-person care but as gateways to dialogue. At the same time, face-to-face interactions reinforced the trust and mutual understanding that had developed online [3,4]. Through this integrated approach, the intervention sought to create a flexible and inclusive health dialogue ecosystem responsive to the realities of rural life (Table 1).

**Table 1:** Components and design principles of the hybrid digital-physical health dialogue model. This table summarizes the core components of the hybrid intervention, their operational characteristics, guiding design principles, and intended functions within the overall health dialogue ecosystem. The digital component (LINE-based health consultation) emphasizes low-threshold access, anonymity, and flexibility to reduce psychological and geographic barriers to help-seeking. The face-to-face component (community center dialogues) focuses on embodied interaction, mutual recognition, and trust-building through horizontal communication. The table also highlights the roles of healthcare professionals and the community-based organization in facilitating dialogue, ensuring community embeddedness, and maintaining feasibility. Together, these components form an integrated hybrid ecosystem designed to support accessible, relational, and sustainable health dialogue in a rural setting. NPO: non-profit organization.

Component	Description	Design principles	Intended function
LINE-based health consultation	Anonymous health consultation provided via the LINE messaging platform, accessible free of charge to community residents	Low-threshold access; anonymity; confidentiality; flexibility	To enable early health dialogue and reduce psychological and geographic barriers to help-seeking
Community center face-to-face dialogues	In-person dialogue sessions conducted at local community centers with residents and healthcare professionals	Embodied interaction; mutual recognition; trust-building; horizontal communication	To foster relational trust, shared understanding, and sustained engagement through direct interaction
Hybrid digital-physical integration	Bidirectional linkage between digital consultations and face-to-face dialogues within a single participatory system	Continuity; adaptability; inclusiveness	To allow flexible movement between digital and physical spaces and support diverse participation patterns
Healthcare professionals	Physicians and other healthcare providers participating as facilitators rather than authoritative clinicians	Supportive facilitation; responsiveness; contextual sensitivity	To provide guidance, validate concerns, and connect dialogue with appropriate healthcare pathways
Community organization (NPO)	Community-based organization coordinating logistics, outreach, and collaboration with healthcare institutions	Community embeddedness; coordination; sustainability	To maintain operational feasibility, strengthen community trust, and support long-term implementation
Hybrid health dialogue ecosystem	Integrated system combining digital and physical components supported by healthcare and community actors	Accessibility; relational depth; feasibility	To create a sustainable, inclusive platform for ongoing health dialogue in a rural setting

Implementation process

**Figure 2:**
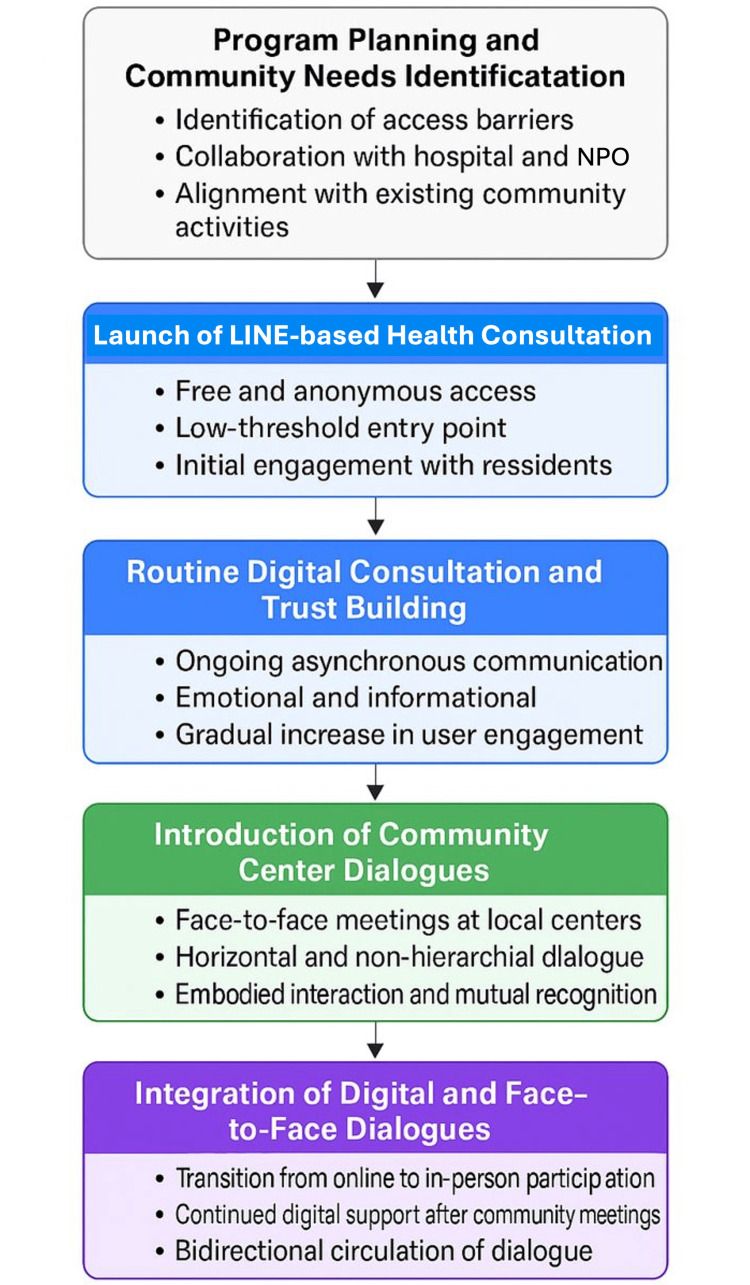
Implementation flow of the hybrid digital-physical health dialogue model. This figure outlines the stepwise implementation process of the hybrid digital-physical health dialogue intervention in a rural community. The process began with the identification of local barriers to healthcare access and help-seeking, followed by the launch of an anonymous, low-threshold LINE-based consultation service. Routine digital consultations enabled early engagement and trust-building among residents. Subsequently, face-to-face dialogue sessions were introduced at community centers to foster embodied communication and collective reflection. Over time, digital and physical dialogue spaces were intentionally integrated, allowing bidirectional participation and sustained engagement. The model was continuously refined through informal feedback and adaptation, facilitating integration into routine community and healthcare practice rather than functioning as a time-limited project. NPO: non-profit organization. Image credit: Ryuichi Ohta.

The hybrid digital-physical health dialogue intervention was implemented through close collaboration between the Unnan Community Health and Development Lab, the Department of Family Medicine at Unnan City Hospital, and local community stakeholders [3,4]. The implementation process emphasized feasibility within routine practice, minimizing additional burden on healthcare professionals while maintaining accessibility for community residents (Figure 2).

Organizational Structure and Roles

The intervention was coordinated by the Unnan Community Health and Development Lab, a community-based organization partnering with healthcare professionals at Unnan City Hospital [3]. Physicians and other healthcare providers participated voluntarily, contributing to both the digital consultation service and community-based dialogues as part of their broader commitment to community engagement rather than as a formal clinical service [12].

Clear role delineation was established at the outset. Healthcare professionals were responsible for responding to LINE-based consultations, facilitating community dialogues, and providing guidance when medical consultation was deemed necessary. The NPO managed logistical arrangements, including coordinating with community centers, scheduling dialogue sessions, and disseminating information about the program to residents. This division of roles enabled efficient operation while leveraging existing professional and community networks.

Launch and Operationalization

The LINE-based consultation service was launched first, serving as an accessible entry point for residents hesitant to engage with formal healthcare settings [3]. The platform was intentionally kept simple, requiring no prior registration beyond using the LINE application. This design was intended to reduce technical barriers and to support early adoption of the service. Consultations were monitored regularly, and responses were provided promptly to maintain trust and engagement.

Following the establishment of the digital service, face-to-face dialogue sessions were gradually introduced at community centers across Unnan City [4]. These sessions were scheduled in coordination with existing community activities with the intention of enhancing participation and reducing residents’ logistical burdens. Healthcare professionals attended as facilitators, adopting a supportive and non-directive role to encourage open dialogue and mutual learning.

Integration into Routine Practice

To ensure sustainability, the intervention was embedded within existing clinical and community workflows rather than operating as a stand-alone project [3,4]. Digital consultations were managed alongside routine clinical duties, with an emphasis on brief, supportive responses rather than comprehensive clinical assessments. When concerns raised through LINE consultations suggested the need for formal medical evaluation, participants were gently encouraged to seek appropriate care through existing healthcare pathways.

Similarly, community dialogues were designed to complement, rather than replace, conventional healthcare services. Insights gained from these sessions informed healthcare professionals’ understanding of local health challenges, enhancing contextual awareness without adding administrative burden. This pragmatic integration helped prevent overburdening staff and supported long-term feasibility.

Adaptation and Iterative Refinement

The implementation process was iterative and responsive to emerging challenges. Feedback from residents and healthcare professionals was informally incorporated to refine communication styles, session formats, and scheduling. For example, the balance between digital anonymity and relational continuity was continuously adjusted to respect participants’ comfort levels with the aim of supporting ongoing engagement.

Throughout implementation, attention was paid to maintaining low-threshold access and avoiding exclusion. Efforts were made to support participants with limited digital literacy through informal assistance during community dialogues, helping bridge the gap between digital and physical engagement. These adaptive practices allowed the intervention to evolve in alignment with community needs while preserving its core principles [3,4].

Outcomes and observations

Through the implementation of the hybrid digital-physical health dialogue model, several notable outcomes and practical observations were identified [3,4]. These findings are presented as descriptive insights derived from routine operation rather than as formally evaluated effectiveness measures. The insights were drawn from routine documentation, facilitator field notes recorded during and after activities, periodic staff debriefings, and informal participant feedback, without the use of structured observation protocols or formal analytic frameworks (Table 2).

**Table 2:** Observed benefits and challenges. This table summarizes descriptive observations regarding perceived benefits, operational challenges, and practical implications identified during routine implementation of the hybrid digital-physical health dialogue model. These observations were derived from routine documentation, facilitator field notes, staff debriefings, and informal participant feedback, and are presented for implementation insight rather than as formally evaluated outcomes.

Domain	Observed benefits	Challenges and limitations	Implications for practice
Accessibility	Residents were able to seek advice at an early stage through anonymous digital consultation	Digital literacy and device access varied among older adults	Combine digital tools with in-person support to reduce exclusion
Help-seeking behaviors	Increased willingness to discuss mild or ambiguous health concerns	Lack of formal outcome measures limits the assessment of behavioral change	Use low-threshold dialogue as a gateway to appropriate care pathways
Trust and engagement	Gradual trust-building enabled transition from digital to face-to-face dialogue	Sustained engagement depended on continued human resources	Ensure relational continuity while managing staff workload
Community connection	Face-to-face dialogues fostered mutual recognition and peer support	Participation was limited for residents with mobility constraints	Maintain hybrid formats to accommodate diverse participation needs
Operational sustainability	Integration into routine practice enhanced feasibility	Reliance on voluntary staff raised sustainability concerns	Explore ethical and context-sensitive funding mechanisms

Patterns of Engagement

The anonymous LINE-based consultation service functioned as a low-threshold entry point for health-related dialogue. Residents used the platform to raise concerns that ranged from mild physical symptoms and medication questions to psychological distress and caregiving-related challenges. Many consultations reflected uncertainty about whether symptoms warranted formal medical attention, suggesting that the service addressed an unmet need for early, informal guidance.

A recurring pattern observed was gradual engagement over time. Some users initially submitted brief, tentative messages and subsequently developed more detailed communication as trust was established. This progression suggested that anonymity and accessibility may have facilitated sustained interaction, particularly among individuals hesitant to seek conventional care.

Transition From Digital to Face-to-Face Dialogue

A crucial observational outcome was the transition of some participants from digital consultations to in-person community dialogues [4]. After repeated interactions via LINE, a subset of participants who had initially avoided face-to-face engagement later attended community center sessions. This pattern was noted through facilitator field notes and informal conversations during routine operations. It may reflect increasing familiarity with the program rather than a formally measured change in confidence or trust. These observations suggest that digital platforms may function as preparatory spaces for subsequent in-person engagement, without implying any evaluation of effectiveness.

Conversely, participants who first encountered the program through community dialogues frequently continued health-related discussions through the LINE platform. This bidirectional movement supported the continuity of engagement beyond time and physical attendance constraints, reinforcing the complementary roles of digital and physical components.

Perceived Impact on Help-Seeking Behavior

Although no formal outcome measures were applied, healthcare professionals involved in the intervention observed changes in residents’ help-seeking behaviors. Participants appeared more willing to discuss symptoms at an earlier stage and to seek clarification regarding appropriate care pathways, based on clinicians’ routine documentation and facilitator reflections during service operation. In several instances, individuals who might otherwise have delayed consultation were encouraged to access formal medical services following digital or in-person dialogue.

From the clinicians’ perspective, these interactions provided insight into residents’ lived experiences and contextual factors influencing health behaviors. This enhanced understanding supported more patient-centered communication during subsequent clinical encounters, suggesting indirect benefits for routine care [3,4].

Community-Level Observations

At the community level, face-to-face dialogues appeared to be associated with shared understanding and mutual support among participants. Based on facilitator field notes and informal participant interactions during routine sessions, residents exchanged experiences related to aging, chronic illness, and caregiving. These exchanges were observed to coincide with expressions of collective awareness and informal peer support, and in some cases with locally initiated health-related activities.

Healthcare professionals reported, through routine documentation and staff reflections, increased awareness of community priorities and concerns that were not readily visible within clinical settings. Taken together, these observations suggest that dialogue-based interventions may help reveal latent community health needs and may support relationship-building between healthcare systems and rural communities, rather than demonstrating evaluated effects [4].

Interpretation of Observations

Taken together, these observations suggest that the hybrid model may support accessible, sustained, and context-sensitive health dialogue in a rural setting. While these impressions do not constitute evidence of effectiveness, they offer practical, implementation-focused insights into how digital and physical platforms might be integrated to support early engagement, trust-building, and adaptive help-seeking behaviors [3,4]. A formal evaluation would be required to confirm these preliminary observations and assess effectiveness. These observations informed subsequent reflection on the intervention's strengths and limitations and guided considerations for future development.

## Discussion

Challenges and limitations

Despite the practical insights gained from implementing the hybrid digital-physical health dialogue model, several challenges and limitations were identified [3,4]. These issues highlight important considerations for interpreting the observations and for guiding future development of similar interventions [13,14].

Digital Divide and Accessibility

Although the LINE-based consultation service lowered psychological and geographic barriers to health dialogue, digital access remained uneven. Some older adults faced difficulties related to digital literacy, device availability, or familiarity with messaging applications. These barriers were observed to limit participation among some residents during routine operation, based on facilitator field notes and staff reflections. In addition, drawing on prior literature, they highlight the broader risk that digital interventions may inadvertently reproduce existing inequities rather than fully resolve them [4]. While informal support during community dialogues partially mitigated this challenge, digital exclusion remained an ongoing concern.

Human Resource Constraints

The intervention relied heavily on the voluntary involvement of healthcare professionals and community staff. Balancing digital consultations and community dialogues with routine clinical responsibilities was reported by staff as challenging in terms of time and workload [15]. Although efforts were made to integrate activities into existing workflows, the long-term sustainability of the model was perceived as contingent on continued professional commitment rather than on formal assessment [16]. While overt fatigue was not systematically measured, the absence of dedicated staffing or institutional support was recognized as a potential risk for participant and provider fatigue, consistent with prior reports [3,4].

Lack of Formal Outcome Evaluation

This technical report describes an implementation experience and observational outcomes rather than a formally designed effectiveness study. No standardized quantitative measures were used to assess changes in health outcomes, help-seeking behaviors, or quality of life. As a result, causal inferences about the intervention's impact cannot be drawn. The absence of comparative data or long-term follow-up further limits the generalizability of the findings.

Context-Specificity and Transferability

The intervention was implemented in a single rural municipality with specific demographic, cultural, and institutional characteristics. Several contextual factors shaped how the intervention was implemented in practice. For example, existing relationships between healthcare professionals and community organizations enabled the coordination of face-to-face dialogue sessions within routine community activities. In contrast, local community networks facilitated informal outreach and participation. In addition, the availability of a community-based organization with prior experience in resident engagement supported ongoing coordination and operational feasibility.

While the principles underlying the hybrid model may be transferable, direct replication in other settings may require substantial adaptation to local contexts, particularly regarding existing partnerships, organizational capacity, and community receptiveness [3,4].

Ethical and Sustainability Considerations

Maintaining free, low-threshold access was a core principle of the intervention; however, this approach also introduced challenges for financial and operational sustainability. Reliance on voluntary labor and external support raised concerns about long-term viability [17]. Efforts to explore ethical monetization strategies were ongoing, but the potential impact of cost-sharing mechanisms on accessibility and equity remained uncertain [3,4].

Interpretation of Observations

Given these limitations, the observations presented in this report should be interpreted as context-dependent implementation insights rather than evidence of effectiveness. They are intended to inform future practice-based initiatives rather than to provide definitive conclusions about the impact of interventions.

Future directions

Building on the implementation experience of the hybrid digital-physical health dialogue model, several directions for future development have been identified [3,4]. These directions aim to enhance sustainability, inclusiveness, and practical applicability while preserving the core principles of low-threshold access and community engagement.

Toward Sustainable Operational Models

One of the primary future challenges involves securing sustainable operational resources. While the intervention has relied on voluntary participation and free access, this model may be challenging to maintain in the long term. Future efforts will explore ethical and context-sensitive funding mechanisms, such as voluntary donations, partial subscription models, and partnerships with local governments or academic institutions [18]. Any financial strategy must be carefully designed to avoid creating new barriers to access or discouraging participation among socially or economically vulnerable residents [3,4].

Expansion and Adaptation to Other Settings

The hybrid model may offer a flexible framework that could be adapted to other rural or resource-limited settings. Future initiatives could explore how such a model might function in communities with differing levels of digital infrastructure, cultural norms, or healthcare capacity. Rather than pursuing direct replication, emphasis could be placed on adapting underlying principles-low-threshold entry, relational continuity, and community embeddedness- to local contexts [19]. Sharing implementation experiences across regions may support iterative refinement and collective learning.

Integration With Formal Healthcare Systems

Further integration with existing healthcare systems represents another important direction. While the current model operates alongside routine clinical care, closer alignment with primary care, public health programs, and social services could enhance continuity and responsiveness. Establishing clearer referral pathways and feedback mechanisms may help translate dialogue-based insights into coordinated care while maintaining the non-clinical, supportive nature of the intervention [9,20].

Development of Evaluation Frameworks

Future work should also consider the development of pragmatic evaluation approaches that balance methodological rigor with feasibility. Mixed methods design incorporating qualitative feedback, process indicators, and selected quantitative measures may offer insights into patterns of engagement, perceived benefits, and potential unintended consequences. Such evaluation efforts should be proportionate to the scope of practice-based interventions and aligned with ethical considerations relevant to community settings.

Ongoing Reflexivity and Community Participation

Finally, sustained reflexivity and participatory governance will be essential to the evolution of the intervention. Continuous dialogue with residents, healthcare professionals, and community stakeholders can inform adjustments to program design and implementation. By maintaining an open, iterative approach, the hybrid model can remain responsive to changing community needs while supporting equitable and meaningful participation in health dialogue [3,4].

## Conclusions

This technical report describes the implementation of a hybrid digital-physical health dialogue model in a rural Japanese community, integrating anonymous online consultations with face-to-face community dialogues. The intervention was designed to address barriers to health access and help-seeking by providing low-threshold entry points while preserving the relational strengths of in-person communication. Observational insights from routine operation suggest that this design may offer a way to combine digital and physical platforms in a manner perceived as supportive of early engagement and ongoing health dialogue. However, these impressions do not constitute evidence of effectiveness and would require formal evaluation to confirm. The findings instead offer practical lessons regarding feasibility, integration into routine practice, and the value of flexible participation pathways in rural contexts. The hybrid model is not presented as a definitive solution but as an adaptable framework that may be refined and contextualized in other rural or resource-limited settings. By sharing implementation experiences and reflecting on challenges and limitations, this report aims to contribute to ongoing discussions on how community-based and digitally supported approaches might enhance accessibility, inclusiveness, and sustainability in rural healthcare.
